# Knockdown of Rab5a expression decreases cancer cell motility and invasion through integrin-mediated signaling pathway

**DOI:** 10.1186/1423-0127-18-58

**Published:** 2011-08-17

**Authors:** Shan-shan Liu, Xiang-mei Chen, Hong-xia Zheng, Shu-liang Shi, Yu Li

**Affiliations:** 1 Department of Life Science and Engineering, Harbin Institute of Technology (HIT), Harbin 150001, P.R. China; 2 Bio-X Center, The Academy of Fundamental and Interdisciplinary Science, Harbin Institute of Technology, Harbin 150080, P. R. China; 3 Department of Medical Microbiology, Peking University Health Science Center, Peking 100191, P. R. China

## Abstract

**Background:**

Rab GTPases function as modulators in intracellular transport. Rab5a, a member of the Rab subfamily of small GTPases, is an important regulator of vesicle traffic from the plasma membrane to early endosomes. Recent findings have reported that Rab5a gene was involved in the progression of cancer. In the present study, we investigated the effect of Rab5a on cervical cancer invasion and metastasis and the molecular mechanism underlying the involvement of Rab5a.

**Methods:**

Rab5a expression was assessed by immunohistochemical analysis on a cervical cancer tissue microarray. RNA interference (RNAi) was performed to knock down the endogenous expression of Rab5a gene in HeLa and SiHa cells. Cell motility was evaluated using invasion assay and wound migration assay *in vitro*. The expression levels of integrin-associated molecules were detected by Western blot and immunofluorescence.

**Results:**

We found that Rab5a was expressed at a high level in cervical cancer tissues. Silencing of Rab5a expression significantly decreased cancer cell motility and invasiveness. The down-regulation of integrin-associated focal adhesion signaling molecules was further detected in Rab5a knockdown cells. Meanwhile, active GTP-bound Rac1, Cdc42, and RhoA were also down-regulated, accompanied with the reduction in the number and size of filopodia and lamellipodia.

**Conclusions:**

Taken together, these data suggest that Rab5a functions in regulating the invasion phenotype, and we propose that this regulation may be via integrin-mediated signaling pathway in cervical cancer cells.

## Background

Cancer is a leading cause of mortality worldwide. Invasion and metastasis is the main biological characteristics of cancer cells, and the major cause of death in patients with cancer. The investigation concerning tumor cell invasion and metastasis has become the focus of intense researches. However, the molecular mechanism of the progression of cancer cell invasion and metastasis has not been fully elucidated.

Small GTPases of the Ras superfamily function as molecular switches that involve in the control of a large variety intracellular processes, including proliferation and differentiation, gene expression, signal transduction, vesicle trafficking, nuclear assembly, and reorganization of cytoskeleton. Most of these small GTPases cycle between two forms: an active GTP-bound form and an inactive GDP-bound form. GTPase activating proteins (GAPs) promote the GDP-bound state of small GTPases by activating their intrinsic GTPase activity, while GTP exchange factors (GEFs) promote the active GTP-bound state by facilitating the exchange of GDP for GTP. So far more than 100 members of small GTPases have been identified in eukaryotes. According to their sequence ho mology, these proteins are classified into five groups: the Ras, Rho/Rac/Cdc42, Sar1/Arf, Rab and Ran subfamilies [1,2]. The Rab GTPases subfamily regulates intracellular vesicle transport, including receptor-mediated endocytosis, exocytosis, and receptors recycling [3]. Emerging evidence shows aberrant expression of the Rab GTPases in many human diseases including cancer [4-6]. Rab5a, a member of the Rab GTPases subfamily, is mainly localized to the cytosolic face of plasma membrane, early endosomes, and clathrin-coated vesicles. It is a key regulator of intracellular vesicle traffic from the plasma membrane to early endosomes [7,8]. Recent findings have revealed that the overexpression of Rab5a gene was correlated with the metastatic potential and malignant degree of lung and stomach cancer [9,10]. It was also reported that Rab5a was involved in EGF signaling pathway and migration in hepatocellular carcinomas [11]. Moreover, Rab5a was proved to be required for the activation of Rac, a member of the Rho GTPases subfamily, and involve in the regulation of actin cytoskeletal organization [12,13].

It is well known that cell to cell and cell to extracellular matrices (ECM) adhesions affect morphological changes involved in cell migration. Integrins, a large family of cell adhesion glycoproteins, mediate the adhesion of cells to ECM and provide traction for cell motility. The heterodimerization of 19 α-subunits and 8 β-subunits paired to yield 25 different integrins, which form transmembrane receptors for a series of ECM molecules [14]. Each pair of integrin αβ heterodimers has defined extracellular ligands. Meanwhile, many proteins present at the cytoplasmic side of focal adhesions are considered to link transmembrane receptors to the actin cytoskeleton. They are classified into two groups: one is structural proteins, including talin, vinculin, α-actinin, and tensin etc; the other is regulating molecules, including paxillin, focal adhesion kinase (FAK) etc. Moreover, integrins also involve in many cellular processes such as gene transcription, proliferation, signal transduction, and in the progression of diseases such as cancer. In fact, several integrins have been proved to show an increased expression pattern in metastatic cancers [15,16]. It is clear that endocytic trafficking could control cell adhesion events, and then affect cell motility [17-19]. However, the link between Rab5a and integrin-mediated signaling pathway and the exact roles of Rab5a in cancer progression remain unclear.

In the present study, we investigated the mechanism of Rab5a involving in the progression of cervical cancer cells invasion and metastasis. The results showed that Rab5a expression was up-regulated in cervical cancer as compared with paired non-tumorous tissues. Moreover, the absence of Rab5a expression reduced the levels of integrin-mediated signaling molecules in cervical cancer cells, thereby decreased cancer cell motility and invasiveness.

## Methods

### Cell culture

HeLa and SiHa cells (human cervical carcinoma cell lines) were maintained in DMEM medium supplemented with 10% fetal calf serum, 2 mM L-glutamine, 100 IU/ml penicillin and 100 μg/ml streptomycin (all from Invitrogen) at 37°C in a humidified atmosphere of 5% CO_2_.

### Antibodies and reagents

Antibodies against Rab5a, Rac1, Cdc42, GFP, FAK, paxillin, vinculin, GAPDH were obtained from Santa Cruz Biotechnology, Inc. Antibodies against integrin-β1, p-FAK (Tyr 397), p-paxillin (Tyr 118) were obtained from Abcam plc. Linear polyethylenimine (PEI, MW~25000) was obtained from Polysciences, Inc. Mitomycin C, glutaraldehyde, and paraformaldehyde were purchased from Sigma-Aldrich. FITC-labeled phalloidine and DAPI were obtained from Invitrogen, Inc. Glutathion-sepharose beads were obtained from GE Healthcare.

### Immunohistochemistry

The detection of Rab5a protein expression was performed with paraffin-embedded cervical cancer tissue array (Shanghai Outdo Biotech). The slide was dewaxed in xylene, and rehydrated through graded alcohol to distilled water. For antigen retrieval the slide was placed in citrate buffer by heating. Then the slide was immersed in 3% H_2_O_2 _for 10 min to quench endogenous peroxidase, and blocked with 10% rabbit serum in PBS for 30 min. The primary antibody was 1:50 diluted in 1% BSA in PBS and incubated with the samples at overnight 4°C. Then the slide was rinsed in PBS, and the HRP-conjugated secondary antibody was applied at 37°C for 30 min. After rinsing with PBS, the section was incubated with DAB for 3 min. Then the slide was counterstained with 20% hematoxylin, dehydrated in graded ethanol, cleared in xylene and mounted with permount.

### siRNAs preparation and cell transfection

Two siRNAs molecule targeting human Rab5a gene (Genbank accession number NM_004162) were designed using the software on Ambion website (siRNA Target Finder) and correspond to the sequences 5'-CCAACCAGGAATCAGTGTT-3' and 5'-CCACAAAATCCAGGAGCAA-3'. A non-targeting random siRNA severed as the negative control, the sequence is 5'-ACTACCGTTGTTATAGGTG-3' (Ambion).

Cells were stably transfected with the negative control siRNA construct (pSilencer4.1-control), or Rab5a-siRNAs plasmids (pSilencer4.1-Rab5a-siRNAs) using linear PEI (MW~25000) respectively. Then the cells were cultured for 3 weeks in 600 μg/ml G418 (Merch).

### Wound migration assay

Cells were plated at 5×10^5 ^cells/well in 12-well dishes and incubated overnight yielding confluent monolayers for wounding. Before an injury line was made using a pipette tip, cells were pretreated with mitomycin C (25 μg/ml) for 45 min. Then cells were allowed to migrate in complete DMEM medium, and photographs were taken immediately and 16 hr after wounding. Results were presented as migration index: the distance migrated by Rab5a-siRNAs cells or Control cells relative to the width of injured line of Control at 0 hr.

### Invasion assay *in vitro*

*In vitro *invasion assay was performed in Matrigel Invasion Chamber with 8 μm pores (Becton Dickinson). Cells were seeded in the upper chamber at 1.2×10^5 ^cells/insert. The lower chamber was filled with complete medium. After incubating for 24 hr, cells were fixed with methanol and stained with 0.1% crystal violet. Count the cells that migrated to the lower side of the membrane under a microscope. Three inserts were assayed for each sample and fifteen fields were counted for each insert.

### Scanning electron microscopy (SEM)

To observe cellular surface, cells were transferred to round coverglasses and incubated overnight. Cells were fixed in PBS containing 2.5% glutaraldehyde and 4% paraformaldehyde for 1 hr. Then samples were rinsed with PBS and postfixed in 1% OsO_4 _for 1 hr, dehydrated in a graded series of ethanol, transferred into acetone, and dried in K850 critical point drier (Emitech). Dried samples were coated with a thin layer of gold in SCD-005 supercoator (BAL-TEC) for 180 sec, and were examined under a Quanta 200 scanning electron microscope (FEI) at 10 kV.

### Confocal laser scanning microscopy

Cells were cultured on round coverglasses for 24 hr (approximately 60% confluence). Then cells were fixed in PBS containing 4% paraformaldehyde for 30 min, permeabilized with 1% Triton X-100 in PBS for 10 min, washed three times with PBS, and blocked in PBS containing 3% BSA for 1 hr. Cells were incubated with primary antibodies diluted in blocking buffer for 1 hr. Cells were then rinsed three times with PBS containing 0.1% Tween-20 (PBST) and incubated with secondary antibodies. DNA was identified by staining with DAPI for 1 min. The coverglasses were then mounted and examined using a LSM 510 META confocal microscope (Zeiss).

### GST-pull down assay of GTP-bound Rac1, Cdc42, and RhoA

GST-pull down assays of active GTP-bound Rac1, Cdc42, and RhoA were performed as described previously [20] with slight modification. GST-RBD (Raf Ras binding domain) and GST-TRBD (Rhotekin Rho binding domain) were expressed by pGEX-RBD and pGEX-TRBD (gift from Dr. Xiang-Dong Ren) in *E. coli *strain *Rosetta (DE3) *respectively and purified on glutathion-sepharose beads. Cells were lysed in 50 mM Tris-HCl, pH 7.4, containing 1% Triton X-100, 150 mM NaCl, 5 mM MgCl_2_, 1 mM DTT, 10 μg/ml aprotinin, 10 μg/ml leupeptin, and 1 mM PMSF. Equal amount of cell lysates were incubated with GST-RBD or GST-TRBD beads for 60 min at 4°C. GTP-bound Rac1, Cdc42, and RhoA were detected by Western blotting. The amount of GTP-bound Rac1, Cdc42, and RhoA was normalized to the total amount of these GTP-bound GTPases in cell lysates in each sample separately.

## Results

### The expression of Rab5a protein is up-regulated in cervical cancers

**Figure 1:**
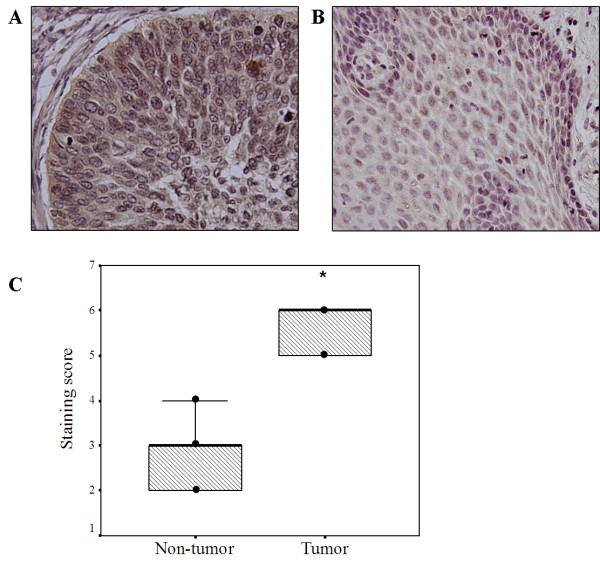
**Immunohistochemical staining of Rab5a protein in cervical cancer and paired adjacent non-tumor tissues**. The expression of Rab5a protein is up-regulated in cervical cancer tissues (A), as compared to their paired non-tumor tissues (B). ×400. (C) Plot showing staining scores on tissue microarray for both tumor tissues and paired non-tumor tissues. *P < 0.05, as determined by Wilcoxon signed-rank test.

To determine the expression pattern of Rab5a protein in cervical cancer, we analyzed Rab5a expression by immunohistochemistry in primary cervical cancer tissues and paired adjacent non-tumorous tissues from 31 patients (62 tissue cores). Rab5a immunostaining was observed to mainly localize in the cytoplasm and membrane of cancer cells. Qualitation of the staining was determined by the percentage of cells showing positive immunoreactivity (0, no staining; 1, <20%; 2, 20-50%; and 3, >50% of cells), and the intensity of staining (graded 0, negative; 1, weak; 2, moderate; and 3, strong). As shown in Figure 1, Rab5a protein levels observed in tumor were higher than those observed in adjacent non-tumorous tissues (*P *< 0.05). The results indicate that Rab5a may be involved in the progression of cervical cancer.

### Knockdown of Rab5a expression decreases cancer cell motility and invasiveness

**Figure 2:**
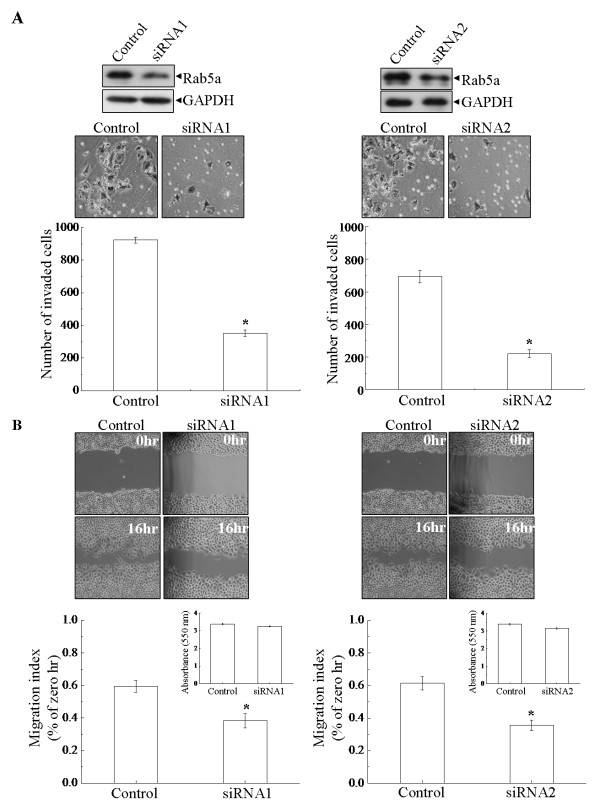
**Knockdown of Rab5a decreases HeLa cell motility and invasion**. (A) In Control and Rab5a-siRNAs cells, the expression of Rab5a was confirmed, and the number of the cells that invaded through the matrigel-coated membrane was determined by counting the cells on the lower side of the membrane under a light microscopy (×200). (B) In Control and Rab5a-siRNAs cells, cell motility was examined with a light microscopy (×40) at the indicated time points, and the wounding width was quantified. MTT data were shown in insets. The data shown represent the mean ± S.E. (n = 3). *P < 0.05 versus Control cells, as determined by two-tailed Student's t test.

**Figure 3:**
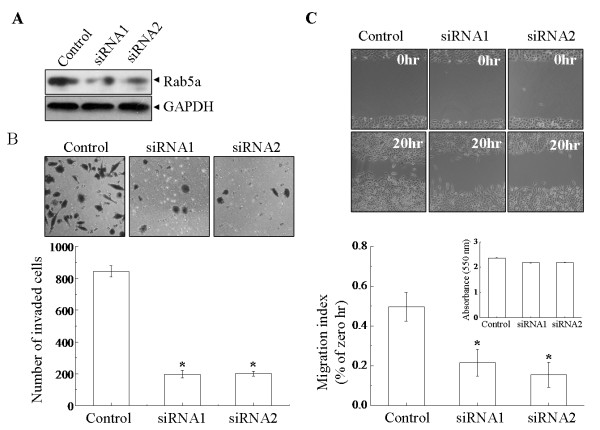
**Knockdown of Rab5a decreases SiHa cell motility and invasion**. (A) In Control and Rab5a-siRNAs cells, the expression of Rab5a was confirmed by Western blot. (B) The number of cells that invaded to the lower side of the membrane was counted under a light microscopy (×200). (C) In Control and Rab5a-siRNAs cells, cell motility was examined with a light microscopy (×40) at the indicated time points, and the wounding width was quantified. MTT data were shown in insets. The data shown represent the mean ± S.E. (n = 3). *P < 0.05 versus Control cells, as determined by two-tailed Student's t test.

To examine whether Rab5a is relative to the progression of cancer cell invasion and metastasis *in vitro*, human cervical carcinoma HeLa and SiHa cells were stably transfected with two siRNAs molecule within the coding sequence of Rab5a gene respectively. Two assays were used to determine the effects of Rab5a knockdown on cell motility: a wound migration assay and an *in vitro *invasion assay using matrigel-coated chamber. Before these assays were performed, Rab5a expression was confirmed firstly (Figure 2A, upper panel and Figure 3A). In the case of invasion analyses, knockdown of Rab5a expression with both siRNAs significantly reduced cell motility (Figure 2A and 3B, P < 0.05). The Rab5a-siRNAs cells that migrated to the lower side of the membrane were threefold lower than Control cells. Wound migration assay also showed that Rab5a knockdown cells had decreased motility, as compared with Control cells (Figure 2B and 3C, P < 0.05). To eliminate the effect of cell proliferation on migration, cells were pretreated with mitomycin C before an injury line was made [21], and MTT assays carried out over the same time periods were presented as insets. These results suggest that the depletion of Rab5a can decrease cancer cell invasiveness and motility *in vitro*.

### Rab5a affects integrin-mediated focal adhesion complex assembly

**Figure 4:**
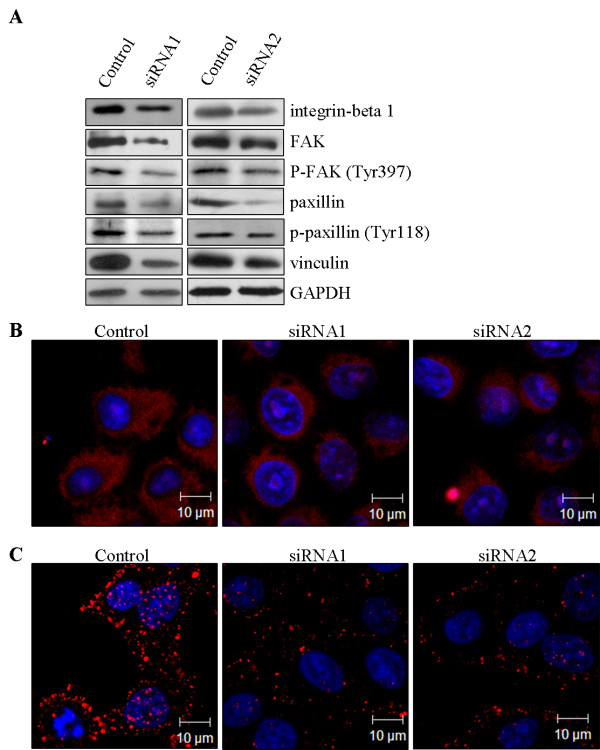
**Rab5a modulates integrin-associated focal adhesion complexes assembly in HeLa cells**. (A) Levels of integrin-mediated focal adhesion proteins in Control and Rab5a-siRNAs cells were detected by Western blot. (B and C) Control and Rab5a-siRNAs cells were stained with integrin β1 (B) and paxillin (C) respectively.

**Figure 5:**
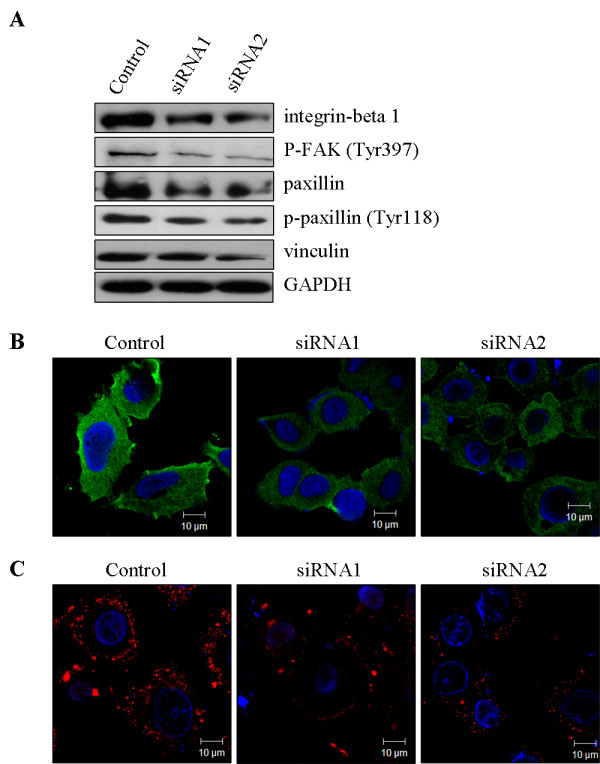
**Rab5a modulates integrin-associated focal adhesion complexes assembly in SiHa cells**. (A) The expression of integrin-associated signaling molecules in Control and Rab5a-siRNAs cells were determined by Western blot. (B and C) Control and Rab5a-siRNAs cells were stained with integrin β1 (B) and paxillin (C) respectively.

Since Rab5a has been identified to involve in cancer cell motility and invasiveness, as described above, we set out to define the effects of Rab5a knockdown on integrin-mediated signaling molecules. We chose to examine the expression levels of integrin β1, FAK, p-FAK (Tyr 397), paxillin, p-paxillin (Tyr 118), and vinculin by Western blot and immunofluorescence. The results showed that all these integrin-mediated signaling proteins, to some extent, were down-regulated in Rab5a-siRNAs cells, as compared with Control (Figure 4A and 5A). As expected, these results were further confirmed by the observation that immunostained integrin β1 and paxillin in Rab5a-siRNAs cells were weaker than Control cells (Figure 4B, C and 5B, C). These data suggest that Rab5a can affect integrin-associated focal adhesion complex assembly.

### Rab5a depletion affects the actin cytoskeletal reorganization

**Figure 6:**
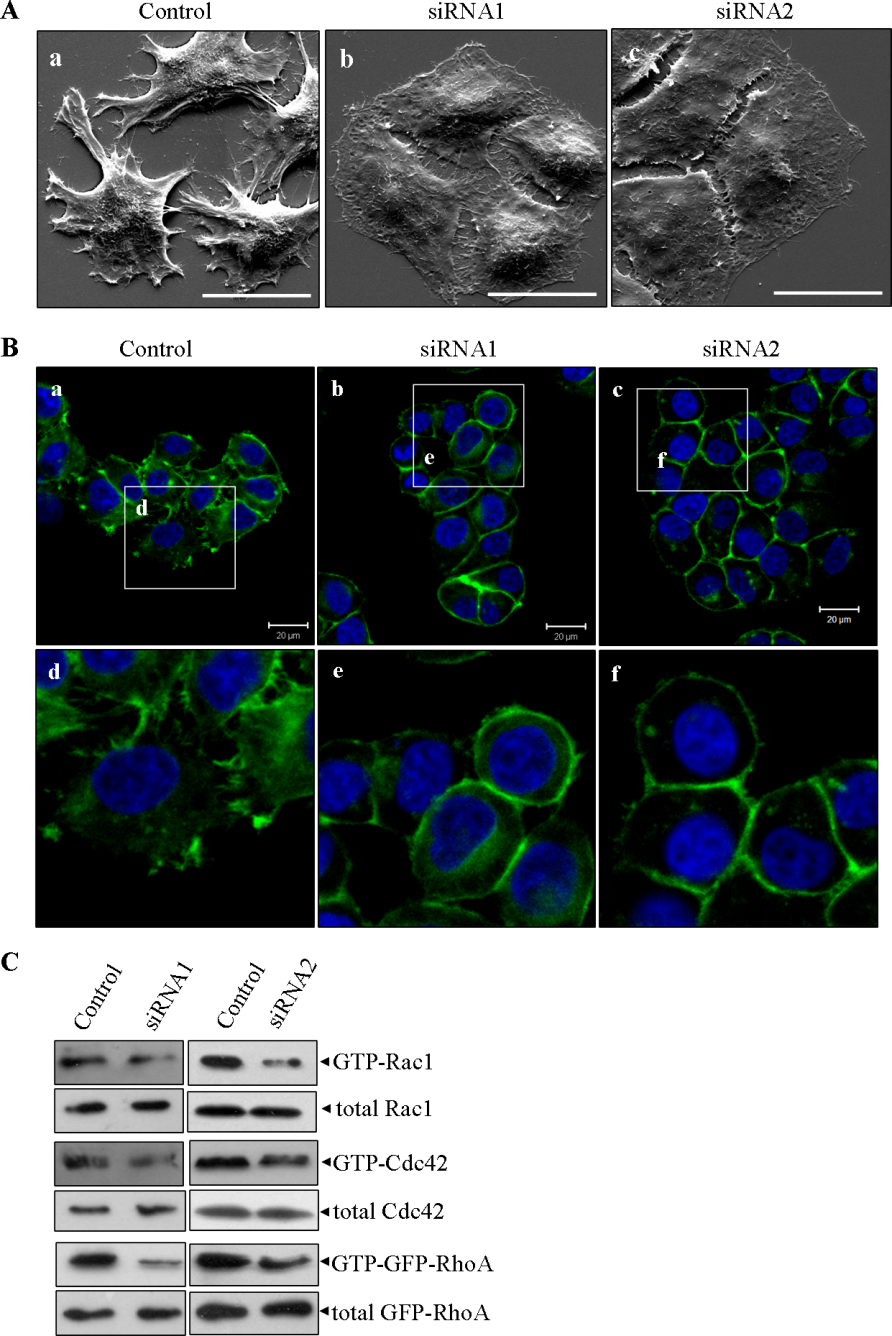
**Rab5a regulates the activity of Rho GTPases and reorganization of actin cytoskeleton in HeLa cells**. (A) The Control and Rab5a-siRNAs cells were fixed and observed by a scanning electron microscopy (SEM). ×5 000. Knockdown of Rab5a expression with both siRNAs (b, c) caused a significant reduction in the number and size of filopodia and lamellipodia formation compared with Control (a). (B) The Control and Rab5a-siRNAs cells were double labeled with DAPI and FITC-phalloidine. Scale bars indicate 20 μm. (C) Levels of active GTP-bound Rac1, Cdc42 and GFP-RhoA in Control and Rab5a-siRNAs cells were detected by GST-pull down assays, as described in the materials and methods. Total Rac1, Cdc42 and GFP-RhoA present in the lysates were analysed by immunoblotting with their specific antibody respectively.

**Figure 7:**
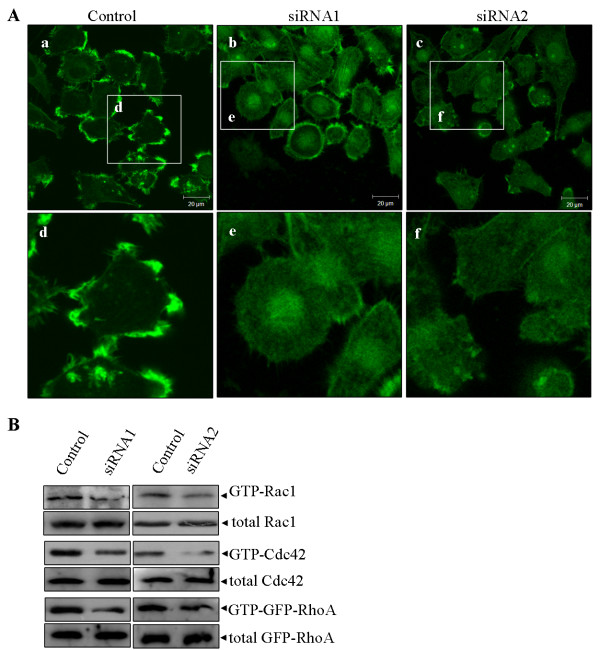
**Rab5a regulates the activity of Rho GTPases and reorganization of actin cytoskeleton in SiHa cells**. (A) The Control and Rab5a-siRNAs cells were stained with FITC-phalloidine. Scale bars indicate 20 μm. (C) Levels of active GTP-bound Rac1, Cdc42 and GFP-RhoA in Control and Rab5a-siRNAs cells were detected by GST-pull down assays, as described in the materials and methods. Total Rac1, Cdc42 and GFP-RhoA present in the lysates were analysed by immunoblotting with their specific antibody respectively.

Since Rab5a has been shown to influent cancer cell invasiveness and mobility, we focus on the effects of Rab5a depletion on the actin cytoskeleton organization. Scanning electron microscopy (SEM) was used to observe the membrane surface ultrastructure of Control and Rab5a knockdown cells. The results showed that knockdown of Rab5a expression with both siRNAs caused a significant reduction in the number and size of filopodia and lamellipodia formation, as compared to Control cells (Figure 6A). Further analyses of phalloidin-stained cells also revealed that the filopodia and lamellipodia formation is obviously inhibited in Rab5a-siRNAs cells (Figure 6B and 7A), which is consistent with the changes on membrane surface ultrastructure detected by SEM. These findings indicate that the absence of Rab5a causes cancer cell morphologic change by action cytoskeleton remodeling.

### Rab5a depletion decreases active GTP-bound Rac1, Cdc42, and RhoA

It is necessary to analyze the effects of Rab5a depletion on the activity of Rho GTPases. GST-pull down assay was performed to detect the active GTP-bound form of Rac1, Cdc42, or RhoA. GST-RBD was used for affinity precipitation of endogenous GTP-bound Rac1 or Cdc42, while GST-TRBD was used to specifically bind GTP-loaded GFP-tagged RhoA because of the lower expression level of endogenous RhoA in HeLa and SiHa cells. The results revealed that the active conformation of GTP-bound Rac1, Cdc42, and RhoA were significantly down-regulated in Rab5a-siRNAs cells (Figure 6C and 7B). These findings implicate that Rab5a has a marked influence on the activation of Rho GTPases. Moreover, these findings explain the observation of actin remodeling in Rab5a-siRNAs cells.

## Discussion

Rab5a is best known as an important regulator of intracellular vesicle transport, while little is known in detail about the pathophysiological role of Rab5a in human diseases. Only a few studies have shown that Rab5a was involved in the progression of cancer. However, the exact role of Rab5a in cancer cell invasion and metastasis is still unknown. In this paper, we confirmed that Rab5a protein was over expressed in cervical cancer compared to the paired non-tumorous tissues. To further identify the role of Rab5a in cancer cell motility and invasiveness, RNAi was used to knock down the expression of endogenous Rab5a in HeLa and SiHa cells. We observed that the depletion of Rab5a not only decreased cancer cell motility and invasiveness, but also inhibited the formation of filopodia and lamellipodia protrusions. These findings suggest that Rab5a may involve in the progression of cancer cell invasion and metastasis by regulating the actin cytoskeleton remodeling. Further studies were mainly focused on the downstream targets of Rab5a.

Cell migration and invasion are essential processes in the metastasis of cancer cells. Migratory cancer cells undergo series of morphological changes by the reconstruction of adhesion and actin cytoskeleton. It is now widely accepted that the process of cancer cells migration mainly consists of four steps: the formation and extension of filopodium and lamellipodium at the leading edge, the establishment of new adhesion sites at the front, contraction of cell body, and detachment of adhesions at the rear [22]. Integrins play a central role in the formation of cell adhesion to ECM. They can form heterodimeric receptors and integrate different extracellular signals to downstream effectors, and then induce cell morphological changes in adapt to the microenvironment. In addition, integrins can influence cell motility not only by providing traction forces to cell migration, but also by regulating actin cytoskeleton remodeling and cell contractility through active Rho GTPases [23]. Rac is responsible for the polymerization of actin to form peripheral lamellipodia protrusions and growth factor-stimulated membrane ruffles, Cdc42 regulates cell polarity and filopodia extension, and Rho triggers stress fibers and cell body contraction [24,25]. Formation of focal adhesions and the closely associated actin stress fibers also requires the activation of the small GTP-binding protein Rho [22,26]. Because integrins have no actin-binding or enzymatic activities, all of downstream signaling events are presumably regulated by proteins associated with their cytoplasmic domains and molecules they recruit [27]. For example, FAK can bind to GTPase-activating protein (GAP) directly, modulate the phosphorylation status of GAPs, and coordinate lamellipodia formation and focal adhesion turnover [28-30]. Paxillin, a focal-adhesion associated adaptor protein, competes with p190RhoGAP for binding to p120RasGAP, and p190RhoGAP freed from p120RasGAP efficiently suppresses RhoA activity during cell adhesion [31,32].

A key element of cell migration is the regulation of adhesive contacts, which are dynamically assembled and disassembled via endocytosis [19]. Adhesion molecules is internalized at the rear and transported to the leading edge, where they become inserted into the membrane to form new adhesions. The depletion of Rab5a influented the internalization and recycling of integrins from the trailing edge to the leading edge, and interrupted the 'outside-in' signaling. The assembly and activities of the downstream molecules were also disrupted, such as the phosphorylation of FAK and paxillin. The balance between GAPs and GEFs was changed, which led to the down-regulation of the active GTP-bound Rho GTPases. Thus, we observed the remodeling of the actin cytoskeleton and impaired cell motility in Rab5a knockdown cells. Taken together, we propose that Rab5a acts as a regulator of integrins and their associated signaling proteins, through which Rab5a can influence the cell's migratory machinery. Therefore, the overexpression of Rab5a in cancer may enhance the integrin-mediated signaling pathway, and induce the cancer cell migration and invasion.

## Conclusions

Here we report that Rab5a involves in the progression of cervix cancer. Rab5a influences cancer cell motility and invasion by regulating the expression levels and activities of integrins and their downstream signaling molecules. Further studies on Rab5a may help us to fully understand tumor invasion and metastasis.

## Competing interests

The authors declare that they have no competing interests.

## Authors' contributions

SSL and SHH designed the study and drafted the manuscript. XMC designed the siRNAs targeted to Rab5a. SSL performed the immunohistochemistry, invasion assay, wound healing assay and GST-pull down assays. HXZ carried out the immunofluorescence assay. SLS helped to draft the manuscript. All authors read and approved the final manuscript.

## References

[B1] Takai Y Sasaki T Matozaki T Small GTP-binding proteins Physiol Rev 2001 81 153 208 11152757 10.1152/physrev.2001.81.1.153

[B2] Lundquist EA Small GTPases WormBook 2006 17 1 18 10.1895/wormbook.1.67.1 PMC4781362 18050472

[B3] Stenmark H Olkkonen VM The Rab family Genome Biol 2001 2 5 3007.1 7 10.1186/gb-2001-2-5-reviews3007 PMC138937 11387043

[B4] Chia WJ Tang BL Emerging roles for Rab family GTPases in human cancer Biochim Biophys Acta 2009 1795 2 110 116 19425190 10.1016/j.bbcan.2008.10.001

[B5] Cheng KW Lahad JP Gray JW Mills GB Emerging role of RAB GTPases in cancer and human disease Cancer Res 2005 65 7 2516 2519 10.1158/0008-5472.CAN-05-0573 15805241

[B6] Zahraour A Touchot N Chardin P Tavitian A The Human Rab Gene5 encode a family of GTP-binding Proteins related to Yeast Ypt1 and SEC4 products involved in secretion J Bio Chem 1989 264 (21): 12394 401 2501306

[B7] Bucci C The small GTPase Rab5 functions as a regulatory factor in the early endocytotic pathway Cell 1992 70 715 28 10.1016/0092-8674(92)90306-W 1516130

[B8] Bucci C Rab5a is a common component of the apical and basolateral endocytic machinery in polarized epithelial cells Proc Natl Acad Sci USA 1994 91 11 5061 5065 10.1073/pnas.91.11.5061 8197185 PMC43931

[B9] Li Y Differential expression of RAB5A gene in human lung adenocarcinoma cells with different metastasis potential Clin Exp Metastasis 1999 17 3 213 219 10.1023/A:1006617016451 10432006

[B10] Li Y Feng HC Chen Y RAB5A, a gene possibly related to metastasis of human carcinoma of the lung and stomach Zhonghua Zhong Liu Za Zhi 1999 21 178 181 11776829

[B11] Koji F Expression of Rab5a in hepatocellular carcinoma: Possible involvement in epidermal growth factor signaling Hepatol Res 2007 37 11 957 965 10.1111/j.1872-034X.2007.00143.x 17581187

[B12] Palamidessi A Frittoli E Garré M Faretta M Mione M Testa I Diaspro A Lanzetti L Scita G Di Fiore PP Endocytic trafficking of Rac is required for the spatial restriction of signaling in cell migration Cell 2008 134 1 135 47 10.1016/j.cell.2008.05.034 18614017

[B13] Lanzetti L Palamidessi A Areces L Scita G Di Fiore PP Rab5 is a signalling GTPase involved in actin remodelling by receptor tyrosine kinases Nature 2004 429 6989 309 14 10.1038/nature02542 15152255

[B14] Hynes RO Integrins: bidirectional, allosteric signaling machines Cell 2002 110 6 673 687 10.1016/S0092-8674(02)00971-6 12297042

[B15] Pellinen T Arjonen A Vuoriluoto K Kallio K Fransen JA Ivaska J Small GTPase Rab21 regulates cell adhesion and controls endosomal traffic of beta1-integrins J Cell Biol 2006 173 5 767 80 10.1083/jcb.200509019 16754960 PMC2063892

[B16] Powelka AM Sun J Li J Gao M Shaw LM Sonnenberg A Hsu VW Stimulation-dependent recycling of integrin beta1 regulated by ARF6 and Rab11 Traffic 2004 5 1 20 36 10.1111/j.1600-0854.2004.00150.x 14675422

[B17] Kawauchi T Sekine K Shikanai M Chihama K Tomita K Kubo K Nakajima K Nabeshima Y Hoshino M Rab GTPases-dependent endocytic pathways regulate neuronal migration and maturation through N-cadherin trafficking Neuron 2010 67 4 588 602 10.1016/j.neuron.2010.07.007 20797536

[B18] Caswell PT Vadrevu S Norman JC Integrins: masters and slaves of endocytic transport Nat Rev Mol Cell Biol 2009 (12): 843 53 10.1038/nrm2799 19904298

[B19] Ulrich F Heisenberg CP Trafficking and cell migration Traffic 2009 10 7 811 818 10.1111/j.1600-0854.2009.00929.x 19490534

[B20] Ren XD William BK Martin AS Regulation of the small GTP-binding protein Rho by cell adhesion and the cytoskeleton The EMBO Journal 1999 18 3 578 585 10.1093/emboj/18.3.578 9927417 PMC1171150

[B21] Kim MS Lee EJ Kim HR Moon A p38 kinase is a key signaling molecule for H-Ras-induced cell motility and invasive phenotype in human breast epithelial cells Cancer Res 2003 63 5454 5461 14500381

[B22] Hall A Raftopoulou M Cell migration: Rho GTPases lead the way Dev Biol 2004 265 1 23 32 10.1016/j.ydbio.2003.06.003 14697350

[B23] Caswell PT Vadrevu S Norman JC Integrins: masters and slaves of endocytic transport Nat Rev Mol Cell Biol 2009 10 12 843 53 10.1038/nrm2799 19904298

[B24] Nobes CD Hall A Rho, rac, and cdc42 GTPases regulate the assembly of multimolecular focal complexes associated with actin stress fibers, lamellipodia and filopodia Cell 1995 81 1 53 62 10.1016/0092-8674(95)90370-4 7536630

[B25] Ridley AJ Hall A The small GTP-binding protein rho regulates the assembly of focal adhesions and actin stress fibers in response to growth factors Cell 1992 70 3 389 399 10.1016/0092-8674(92)90163-7 1643657

[B26] Clark EA King WG Brugge JS Symons M Hynes RO Integrin-mediated signals regulated by members of the rho family of GTPases J Cell Biol 1998 142 2 573 586 10.1083/jcb.142.2.573 9679153 PMC2133065

[B27] Lo SH Focal adhesions: what's new inside Dev Biol 2006 294 2 280 291 10.1016/j.ydbio.2006.03.029 16650401

[B28] Schaller MD Cellular functions of FAK kinases: insight into molecular echanisms and novel functions J Cell Sci 2010 123 1007 1013 10.1242/jcs.045112 20332118

[B29] Chan KT Cortesio CL Huttenlocher A FAK alters invadopodia and focal adhesion composition and dynamics to regulate breast cancer invasion J Cell Biol 2009 185 357 370 10.1083/jcb.200809110 19364917 PMC2700377

[B30] Tomar A Schlaepfer DD Focal adhesion kinase: switching between GAPs and GEFs in the regulation of cell motility Curr Opin Cell Biol 2009 21 676 683 10.1016/j.ceb.2009.05.006 19525103 PMC2754589

[B31] Brown MC Turner CE Paxillin: adapting to change Physiol Rev 2004 84 4 1315 39 10.1152/physrev.00002.2004 15383653

[B32] Tsubouchi A Sakakura J Yagi R Mazaki Y Schaefer E Yano H Sabe H Localized suppression of RhoA activity by Tyr31/118-phosphorylated paxillin in cell adhesion and migration J Cell Biol 2002 159 673 683 10.1083/jcb.200202117 12446743 PMC2173105

